# Assessing the Development and Viability of an Android App for Auditory Training in Older Adults with Hearing Impairments

**DOI:** 10.1007/s12070-023-03696-3

**Published:** 2023-04-19

**Authors:** Sanjana Madhukesh, Archana Gundmi, Harisha K S, Pramath Ramesh, Roshan Jacob

**Affiliations:** 1 Department of Speech and Hearing, Manipal College of Health Professions (MCHP), Manipal Academy of Higher Education , Manipal, India; 2 Department of Computer Applications, Manipal Institute of Technology, MAHE, Manipal, India

**Keywords:** Auditory training, Android application, Older adults, Speech in noise, Hearing loss

## Abstract

**Purpose**: This study aims to develop an android application that is more compatible and user-friendly for the older adult population with hearing impairment and validate the developed app. **Method**: The app’s framework involved a training mode platform consisting of four levels with four sub-levels each. Every level includes stimuli of word-in-noise containing high and low-frequency words with three different noise types – traffic noise, competing for speech, and speech babble. The levels in the app increased in complexity to provide an efficient auditory training feature. The developed application was validated on older adults and professionals based on a questionnaire with both closed and open-ended questions. **Results**: Based on the validated responses of the participants, the app is a reliable tool for auditory training in older adults with hearing impairment. The app contains ease-of-use features for older adults and has been considered a platform for improvement in aural rehabilitation. **Conclusion**: The study marks as a tool in auditory training for older adults to provide the utmost benefit. Increase in the cases of the hearing-impaired population in a few years, a demand to set up a user-friendly management option is beneficiDigitizationtion of aural rehabilitation, especially for older adults, has thus been noted in the present study.

## Introduction

Hearing impairment is known to be one of the highest occurring sensory deficits in the world, accounting for 30% of the population over the age of 55 years. In the Indian context, a similar prevalence is found and comprised of about 56–62% [1]. Hearing loss in older adults affects their communication skills, which results in poor comprehension and expression of language skills. This can further lead to reduced functional ability, cognitive decline, and depression, which are strongly associated with decreased quality of life [2]. The typical clinical symptoms observed secondary to hearing loss are difficulty understanding speech in noise, ringing in the ears, request for repeated speech, and lessons. Based on this critical essential detailed management protocol is necessary.

The main aim of rehabilitative audiology is to overcome the potential handicapping effect and provide good benefits in communication and personal growth [3]. Hearing aids or amplification devices are the most common management mode for individuals with hearing loss. It provides amplified sounds to the auditory system. With developing technology, these devices incorporate various features of noise reduction algorithms, Bluetooth connectivity, assistive hearing technology, and adaptable features. Although hearing aid amplification may improve the awareness of sound sources, meeting the-life environmental situations is not observed [4, 5]. An integrated management profile approach includes amplification devices and auditory training, and/or auditory verbal therapy enhances cognition and quality of life.

Auditory training as a mode of intervention strategy has been examined to improve basic auditory skills and central auditory processing by enhancing the plastic changes in the brain [6]. In standard terms, it involves the process of teaching or stimulating the brain repeatedly through active sounds, words, and sentences, whereby the listeners can make perceptual distinctions between the trained sounds in a systematic order [7].

The training-related improvements support the need to consider auditory training strategies in managing individuals who express concerns about hearing in difficult listening situations [8]. The type of stimulus plays an essential vital stimulating the auditory system. Using speech stimuli, it has been observed improvement in overall speech perception abilities [9]. Speech stimulus with high familiarity has known to provide a been shorter time for response and acts as a primary cue to understand the stimulus [10].triggerition of noise in the training program along with highly familiar words provides a natural setting for the scope of improving communication [11] and enhancing function [12].

There has been a huge demand to create a dynamic and effective mode of digitized aural rehabilitation and other sensory training [13]. Although desktop-based apps are enormous, mobile apps are limited. A potentially beneficial program Angel Sound has been considered a reliable tool for auditory training. Likewise, mobile apps such as Hear Coach and AB Clix have used the features of providing stimulus in the presence of noise. Developing a flexible mobile app for older adults in Indian languages has not been explored much. Kannada is a Dravidian language spoken in the state of Karnataka, and there has been limited evidence of the development of mobile apps which focus on auditory training. A comprehensive management model for older adults with hearing loss for native speakers of Kannada is essential. This shall contribute as a tool in clinical rehabilitation for older adults with hearing impairment to initiate auditory training along with hearing aids or amplification devices.

The present study is primarily addressed the development of a mobile app and validating it. This was to ensure that the app provided portability and flexibility of words in noise implemented in the Kannada language. To address this need, “Shrutham” was developed as free mobile health (mHealth) app to provide clinically relevant auditory training.

## Methodology

The study was approved by the Institute of Research and Ethical Committee (IEC – 153/2019) and Clinical Trial Registry-India (CTRI) (2020/08/027449). It has been studied under two objectives – the development of the android app and the validation of the developed app.

## Development of the Android Application

To record the stimulus of word-in-noise, 600 words, and 300 high- and low-frequency words were selected from a study, Lexical Database in Kannada [14]. These words pertain to the frequency of occurrence, i.e., the more and less frequently occurring words. The three types of noise chosen for the stimulus were competing speech and speech babble [15] along with traffic noise which was measured through a calibrated sound level meter and processed according to standard parameters. The selected word list was recorded with a sampling rate of 44,000 Hz and a time window of 60 min in the Computerized Speech Lab (CSL). The recording was done in a soundproof room with a microphone placed 6 cm away from the speaker. A male and female native Kannada speaker aged 25 to 35 with good intelligibility was selected for recording. The recorded words and noise were merged with the Audacity software version 2.4.2 installed in Windows 10 Asus A555L. Initially, the noise sample and words were imported through the .wav file to the software window for which they change SNR, mainly 0 dB SNR and − 10 dB SNR.

### The Framework of the App and User Interface

**Fig. 1:**
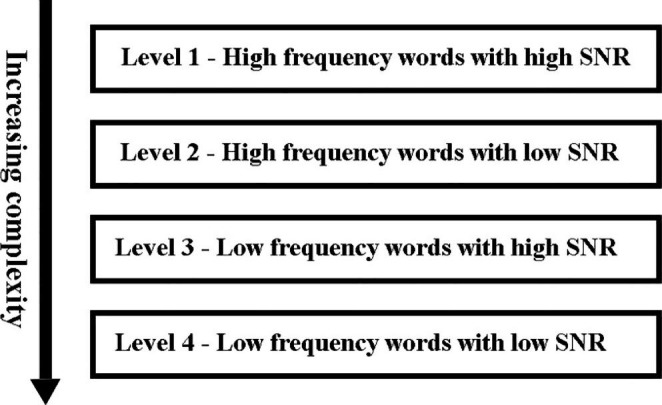
Schematic representation of the auditory training program on the android platform. The framework consists of four levels in increasing complexity concerning the type of words used and varying Signal-to-Noise Ratio (SNR).

**Fig. 2:**
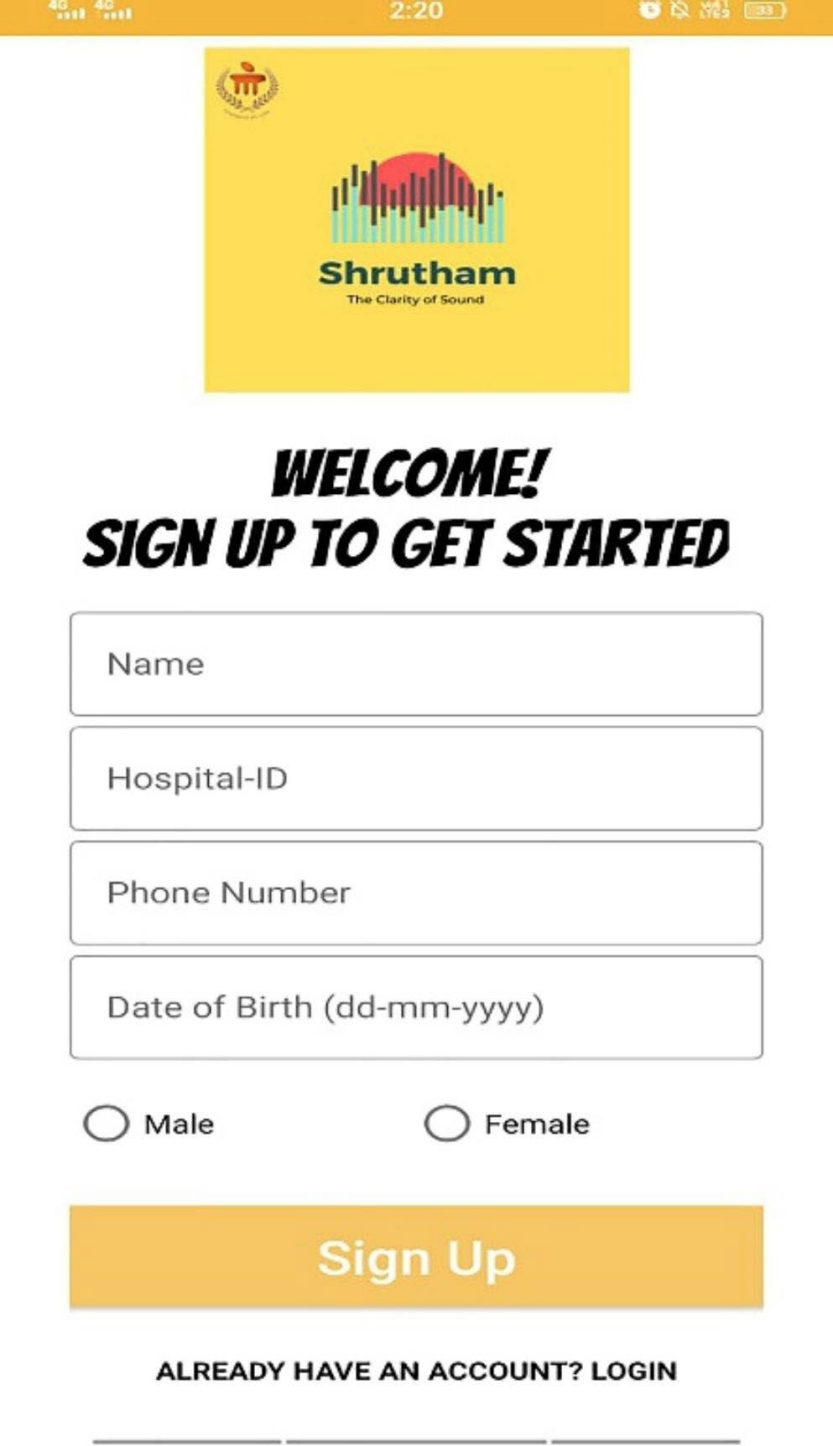
Screenshot of the app representing the screen to create a profile for training

The mobile application is designed to provide auditory training and promote efficient learning through user responses. The interface involves recording user details along with their hospital ID (Fig. 2). Once the details have been submitted, authorization of the user’s phone number is done using Google authentication service by inputting the received One-Time Password (OTP). The interface presents a game-like home screen, having 4 levels with increased complexity and each level having four stages (Figs. 2 and 4). Each level increases in complexity from high to low-frequency words and decreases SNR as shown in Fig. 1. The complexity of each level in the training is based on the amount of varying noise levels (0dB and 10dB SNR) based on the integration of speech in the presence of noise. With increasing noise levels (background noise), the auditory tests tap higher centres of the brain. On the press of the respective stage, a new page is shown, where the stimuli are played to the user, and the response is recorded.

With the help of Google’s speech recognizer Application Programming Interface (API), the application records the user’s response in the native language (Kannada) and matches the response to the voice in the audio (stimuli). On the occasion of the user, not being able to record, or distinguish the noise accurately, he/she is also provided with 4 choices of different words which on selection would indicate whether the option selected is correct/incorrect based upon whether the word in the stimuli is the same or not. Upon the completion of the stage, the user’s tally or score is saved in a database with the help of FIREBASE. The android application has been coded mainly in JAVA while using XML for designing along with FIREBASE as the database in the backend for storage and retrieval. The data stored is further used to carry out analysis on the user’s response and validate the technique fruitfully.

**Fig. 3:**
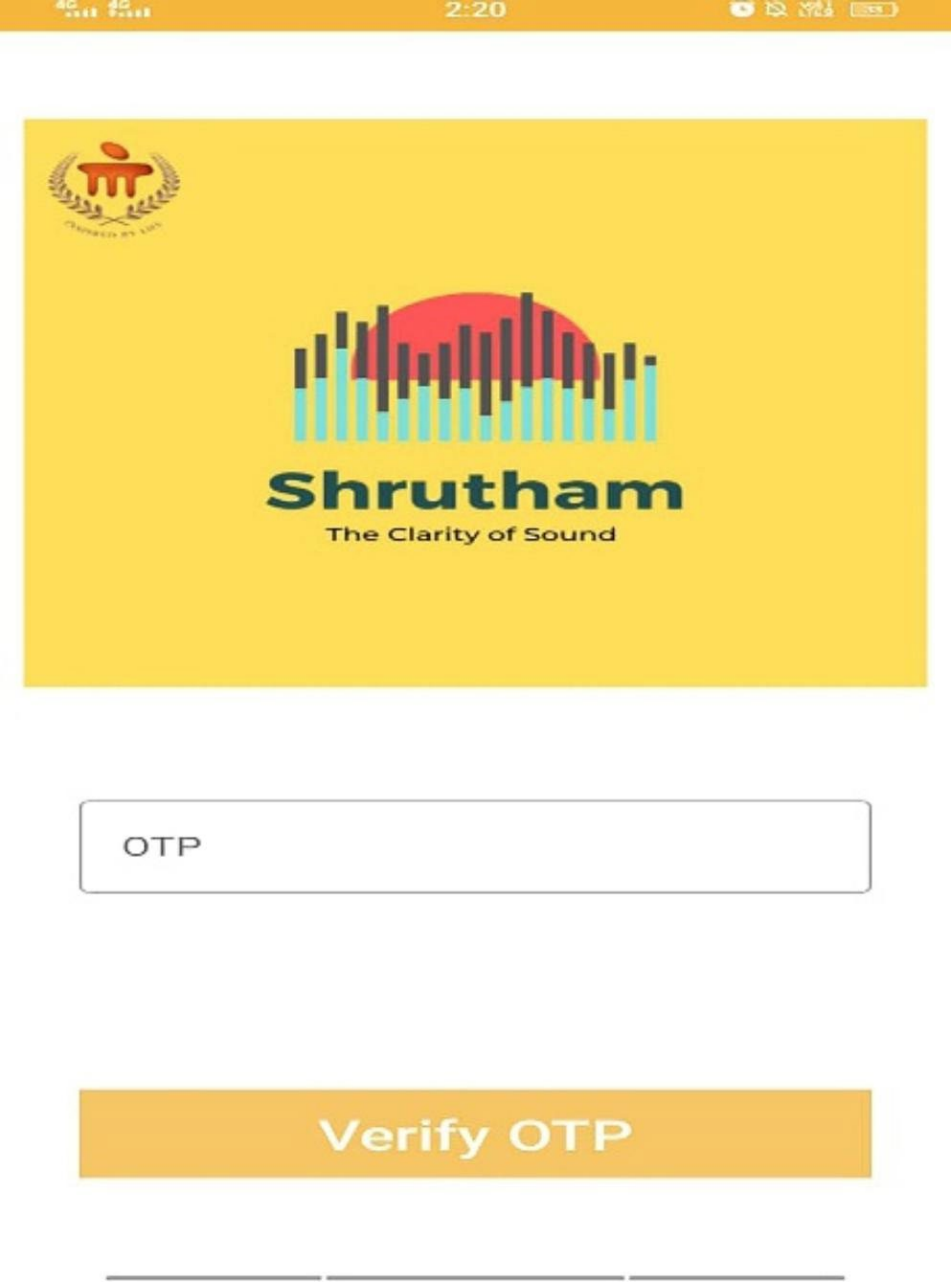
Screenshot of the app representing the screen for an authentication process of OTP.

The application is designed to run only in android as it is coded in JAVA and is limited to the scalability of FIREBASE. The authentication service provided by Google has limits to the number of users and duration of OTP generation (Fig. 3), hence the application makes use of shared preferences to store sessions of the user. The application is versatile in its functionalities, allowing the user to replay the audio or skip to the next audio.

**Fig. 4:**
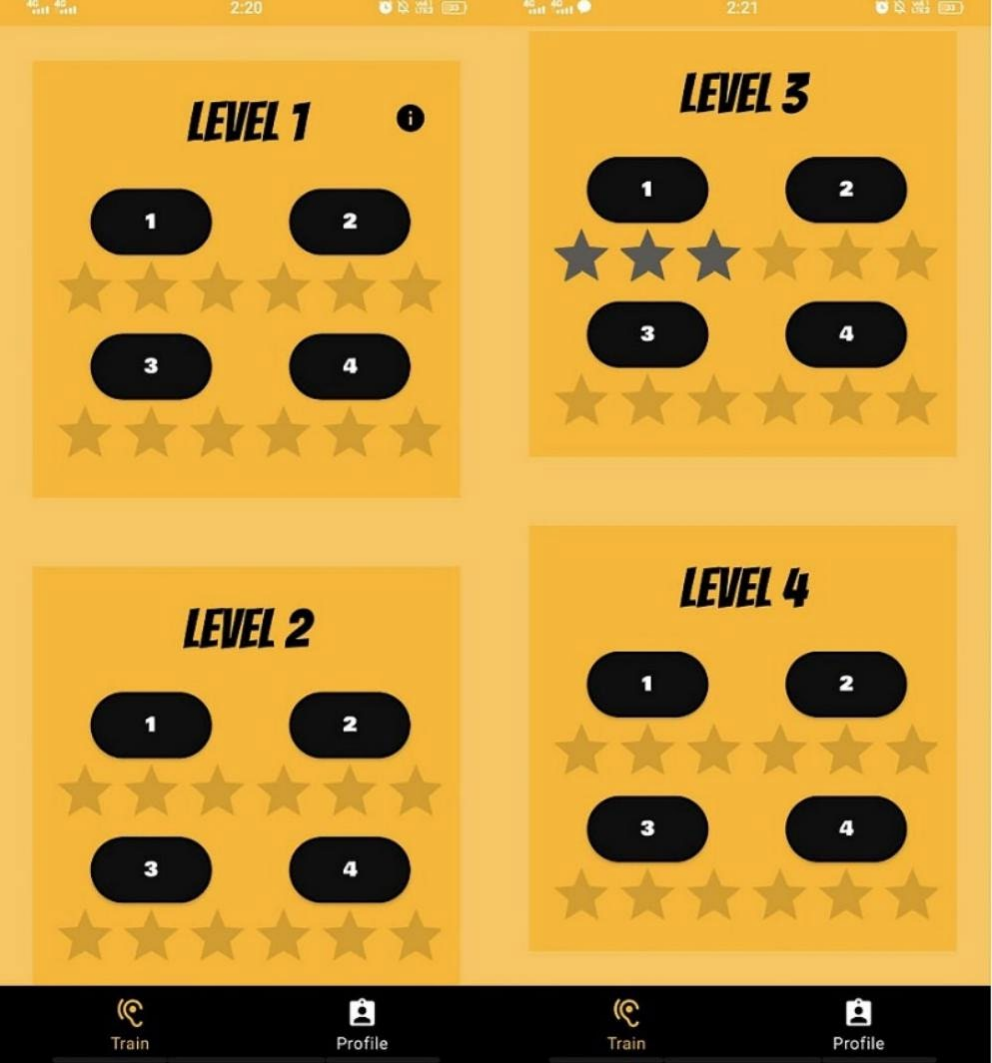
Screenshot of the app representing the four different levels and their stages for auditory training. After completion of the stage, the visual reinforcer is provided (stars)

**Fig. 5:**
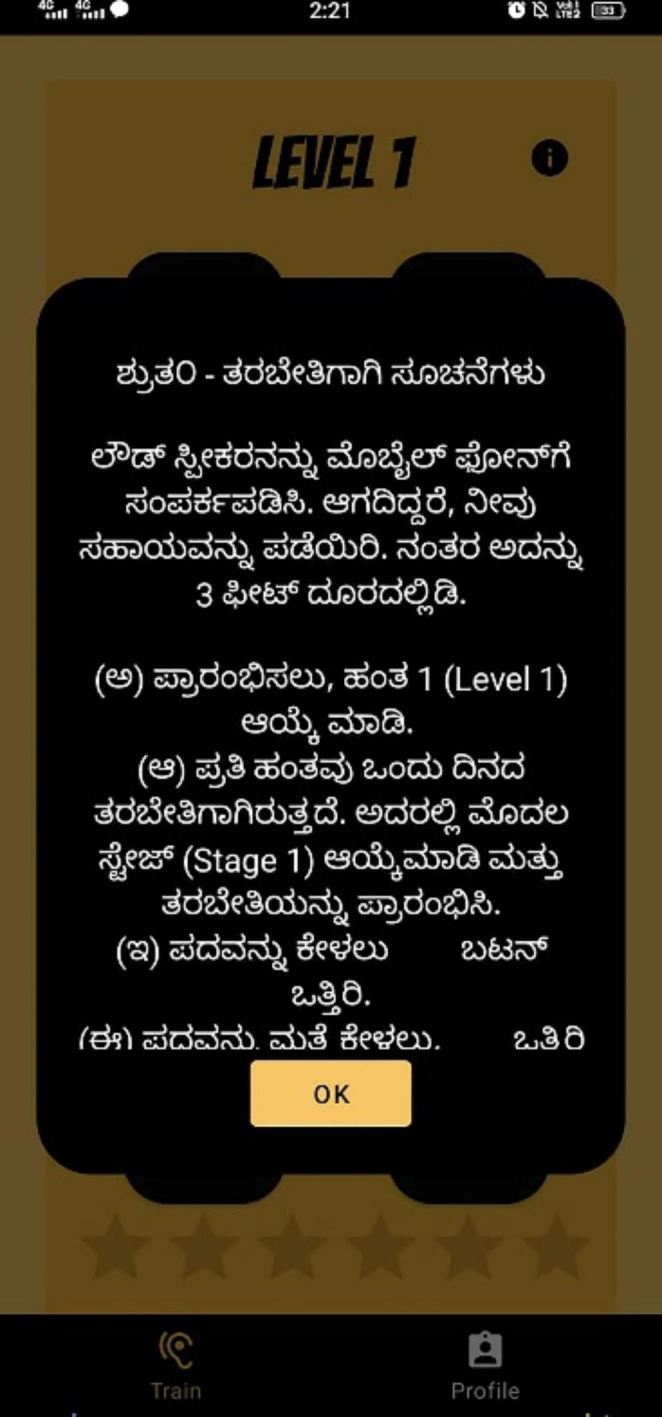
Screenshot of the app representing the instructions for the participants to proceed during the training

## Validation of the Android Application

The process of validation was done with two groups of the population, namely, older adults and professional participants.

### Validation on Older Adults

For the validation of the first set of participants, 20 older adults of age 55 to 75 years of age were recruited. This group consisted of older adults with near-normal hearing and native Kannada speakers. The participants who chose to participate in the study and agreed through consent received a message with instructions to download and install the app on their phone. The other set of participants who chose to participate, met the investigator to directly explore the app and give feedback. While the participants were going through the app, the internal logging-in function would record the app usage data based on the profile and its functions created. Soon after exploring the various features of the app, the participants were sent a feedback form in Kannada which consisted of questions about the usability of the app, ease of use, and an open-ended question for additional comments.

### Validation of Professional Participants

The professionals recruited for the study have at least 5 years of experience in Aural Rehabilitation and Computer Applications. The participants who chose to participate in the study and agreed through consent received a message with instructions to download and install the app on their phone. Similar to the previous validation procedure, the internal logging-in function would record the app usage data based on the profile created and the functions used. Soon after exploring the various features of the app, the participants and professionals were sent an online feedback form. A standard format of System Usability Scale (SUS) developed at the Digital Equipment Corporation and an open-ended question to receive feedback on acoustical and technical aspects.

## Results

### Validation on Older Adults

Descriptive statistics were used to summarize the data from the checklists, both closed and open-ended questions. The results of the usability questionnaire are presented in Table 1.

**Table 1:** Perceived usability of participants (older adults) (n = 20)

Items n (%)	Rating				
	1: Strongly disagree	2: Disagree	3: Neutral	4: Agree	5: Strongly agree
*1. I am willing to use this application frequently*	0 (0.00)	0 (0.00)	1 (5.00)	7 (35.0)	12 (60.0)
*2. I found this application complex*	9 (45.00)	4 (20.0)	0 (0.00)	3 (15.0)	4 (20.0)
*2. I though this application was easy to use*	0 (0.00)	1 (0.00)	0 (0.00)	5 (25.0)	14 (70.0)
*3. I think that I would need assistance to be able to use this application.*	6 (30.0)	2 (10.0)	2 (10.0)	4 (20.0)	6 (30.00)
*4. I found the various functions in this application were well integrated.*	0 (0.00)	1 (5.00)	0 (0.00)	10 (50.0)	9 (45.0)
*5. I would imagine that most people would learn to use this application very quickly.*	1 (5.00)	0 (0.00)	1 (5.00)	6 (30.0)	12 (60.0)
*6. I felt very confident using this application.*	0 (0.00)	0 (0.00)	1 (5.00)	9 (45.0)	10 (50.0)
*7. I needed to learn a lot of things before I could get going with this application*	7 (35.0)	1 (5.00)	3 (15.0)	5 (25.0)	4 (20.0)
*8. I would recommend this application to older adults with Hearing impairment.*	1 (5.00)	0 (0.00)	1 (5.00)	5 (25.0)	13 (65.0)

Overall, all participants strongly agreed that the app was easy to use. The majority of the participants have rated the app to be useful in functions and have found the app less complex. Almost 60% of the participant’s rate that they would be willing to use the application and found that most people would learn to use the mobile app very quickly. Another 65% of the participants strongly agree to recommend the app for older adults with hearing impairment. For the feedback on assistance required, there were mixed responses (40% disagree and 60% agree).

The response to the open-ended question highlighted certain points such as the app being useful and well developed for older adults. With regards to improvement in the app, certain feedbacks also included that the “app stops responding abruptly and captions are needed for symbol understanding”, “More levels can be included and with more varieties” and “More interesting and useful features can be added.“

### Validation of Professional Participants

With the System Usability Scale (SUS) results on professionals, Table 2 delineates the ratings.

**Table 2:** Professional validation of the app (n = 3)

Items n (%)	Rating				
	1: Strongly disagree	2: Disagree	3: Neutral	4: Agree	5: Strongly agree
*1. I am willing to use this application frequently*	0 (0.00)	0 (0.00)	0 (0.00)	1 (33.3)	2 (66.7)
*2. I found this application complex*	0 (0.00)	3 (0.00)	0 (0.00)	0 (0.00)	0 (0.00)
*3. I though this application was easy to use*	0 (0.00)	0 (0.00)	1 (33.3)	1 (33.3)	1 (33.3)
*4. I think that I would need assistance to be able to use this application.*	2 (66.7)	1 (33.3)	0 (0.00)	0 (0.00)	0 (0.00)
*5. I found the various functions in this application were well integrated.*	0 (0.00)	0 (0.00)	0 (0.00)	2 (66.7)	1 (33.3)
*6. There was too much inconsistency in the application.*	0 (0.00)	3 (100.0)	0 (0.00)	0 (0.00)	0 (0.00)
*7. I would imagine that most people would learn to use this application very quickly.*	0 (0.00)	0 (0.00)	0 (0.00)	0 (0.00)	3 (100.0)
*8. I found this application very cumbersome/ awkward to use*	3 (100.0)	0 (0.00)	0 (0.00)	0 (0.00)	0 (0.00)
*9. I felt very confident using this application.*	0 (0.00)	0 (0.00)	0 (0.00)	0 (0.00)	3 (100.0)
*10. I needed to learn a lot of things before I could get going with this application*	2 (66.7)	0 (0.00)	0 (0.00)	1 (33.3)	0 (0.00)

All three professionals have given equal outputs that they would be willing to recommend the app to older adults with hearing impairment and also other professionals. Additionally, a professional gave valuable feedback regarding the app in detailed terms, highlighting that there is ease of handling and a good voice recognition feature. The suggestions were to improve on the theme of the app, fonts, and the overall response patterns and reinforcement to the participants. This provided feedback on the better flow of the content and easier navigation of the app.

## Discussion

World Health Organisation estimates around millions of people with hearing loss within few years and the lack of auditory training is a substantial public health need. With advancements in technology, it will be beneficial to provide auditory training apps to more people with hearing loss. Specifically, in older adults, where communication is hampered, understanding in the presence of noise is substantially preserved and considered to get better with regular training. With advancements in technology with difficult times such as a pandemic, an app on a personal mobile phone will be a model for tele-rehabilitation.

The present study aimed to develop an android app for auditory training in older adults with hearing loss and validate the app through a user feedback questionnaire. Based on the validation process done by older adults and professionals, both groups have given positive reviews on the developed app. Based on the specifications of the app, they have noted that the app has considerable set-up favoring older adults with hearing impairment. Certain features in the mobile app such as response modes like multiple-choice answers, voice recognition, increased complexity of stimulus in training depicted in various levels, and instruction page have been considered as a valid and reliable method of the training application. The response mode of voice recognition and multiple-choice answers serve as an easier option to choose for older adults with hearing impairment. Voice-to-text technology has been supportive for individuals with communication difficulties in the elderly, especially hearing impairment. This feature has been used in a mobile app for teaching hearing-impaired children which proves to be effective for responding to a stimulus [16]. For older adults with sensory issues like vision and dexterity issues, responding through a voice recognition feature can encompass an easier route for using the app. This enables a voice-based interaction in an auditory training program where older adults can reply to new opportunities. Furthermore, with feedback-specific updates in the app, an older adult with hearing impairment will receive a better tool in the area of aural rehabilitation. This platform thus digitizes a validated model of auditory training that uses word-in-noise stimuli with targeted speech sounds.

Another important aspect of the mobile app is, presenting stimulus (words) in presence of noise. This has proven to be effective in the areas of assessment and management [17, 18]. Researchers have shown various methods of enhancing speech perception in the background noise or reverberant conditions, particularly among hearing-impaired individuals [19, 20] and older adults (Bergman, 1980). These studies indicate that perception of speech can be improved with intensive training of words or sentences in various difficult-to-listen conditions [17, 21]. A customized training program, cLEAR™ [22] included word-in-noise training for adults with or without hearing loss. This program enhanced the training benefits for individuals with hearing loss and a need to implement it clinically for aural rehabilitation. Likewise, a long-term training program was studied with words (high and low frequency) and sentences in the presence of speech-shaped noise. The training was provided to older adults of aided and unaided groups. The training resulted in significant betterment in speech perception in noise for an extended period [23]. With respect to an Indian study, similar to Listening and Communication Enhancement (LACE) [24], software was developed in Kannada language to find the efficacy of auditory training. It included various levels of degraded speech and cognitive tasks. The efficacy of training was found to be significantly enhanced in older adults with hearing impairment indicating an improved Signal to Noise ratio [15]. Thus, training in presence of noise will aid the individual to cope up in a realistic environment and enhance cognitive and other sensory abilities.

Adding to the speech-in-noise stimulus, the type of stimulus plays an important role – linguistic and non-linguistic content. Auditory training programs have used different types of stimuli – phonemes, monosyllables, phonetically balanced words, high and low-frequency words, and sentences with target words. Similar to the current study, the use of high and low probability words has been used in other auditory training tasks. With better retrieval capacity, high probability words have obtained better results. It also provides better contextual information and is also stated in the literature that listeners tend to focus on the familiarity of the words during identification of speech in presence of speech babble as a reliable noise type. [25] also witnessed training-related benefits in adults with hearing impairment by using high probability words and further provided evidence that significant changes can be observed with these paradigms. A combination of words of high and low probability along with noise was used in a standardized software named iAngel Sound [26]. This software contains different stimuli for training, among which, high probability sentences in the presence of noise are considered as a stimulus advantageous for auditory training. With these findings, a mobile app using words with high and low familiarity will be beneficial with the hearing-impaired population. Thus, speech perception in noise along with contextual words containing high frequency words provide a better model of auditory training.

## Conclusion

The developed app in the present study provides an insight into rehabilitative audiology, mainly in auditory training for older adults with hearing loss. This marks as a beneficial tool by including various features of Kannada high and low-frequency words along with various noise types, response modes, instruction manual. These create a unique program on an android platform. It has been marked as a reliable, flexible, and useful platform for auditory training. Lastly, with the increased use of technology in audiology and mainly in the areas of telehealth (assessment and management), the use of a digitized network will be a dynamic modification.

## Future Directions

As an effective mode of management in auditory training, the developed mobile app is found to provide sufficient benefit according to older adults and professionals. By adding additional features to the app, it plays a reliable and sensitive tool in the rehabilitation of older adults.

Additionally, to find the efficacy of the app, a group of target population i.e., older adults with Hearing loss and hearing aid users should take part in the study. This enables a higher effect size of the study and the overall clinical relevance of the app can be accounted for in updated versions. The target population can be a part of the training program as a part of a treatment plan and provide sufficient feedback which can be a reliable response.

With advancements in technology in difficult times such as a pandemic, an app on a personal mobile phone for older adults with hearing impairment will be a model for tele-rehabilitation and telehealth programs. This mode of training can also be improvised in different clinical population such as children with hearing loss, generally adults with central auditory processing disorder to train them with speech in noise stimuli.

## References

[1] Malhotra M (2020). The clinical–audiological Cross Sectional Study of Deaf-Mute patients in a Tertiary Care Centre of Uttarakhand State and Literature Review. Indian J Otolaryngol Head Neck Surg.

[2] Somnath A, Gundmi A, Bhargavi P (2020). Comparison of cognitive functions in Elderly Population with and without hearing loss. Indian J Otol.

[3] Hull RH (2019) *Introduction to Aural Rehabilitation: Serving Children and Adults with Hearing loss*, Edition 3. Plural Publishing,

[4] Pasta A, Petersen MK, Jensen KJ, Larsen JE (2019) “Rethinking hearing AIDS as recommender systems,“ *CEUR Workshop Proc*, vol. 2439, pp. 11–17,

[5] Gil D, Iorio MCM (2010) “Formal auditory training in adult hearing aid users,“ *Clinics*, vol. 65, no. 2, pp. 165–174, doi: 10.1590/S1807-59322010000200008 10.1590/S1807-59322010000200008 PMC2827703 20186300

[6] Chermak GD, Musiek FE (2014) Handbook of Central Auditory Processing Disorder: comprehensive intervention. Plural Publishing

[7] Schow RL, Nerbonne (2006). Introduction to Audiologic Rehabilitation.

[8] Anderson S, Kraus N (2013). Auditory training: evidence for neural plasticity in older adults. Perspect Hear Hear Disord Res Diagnostics.

[9] Anderson S, White-Schwoch T, Choi HJ, Kraus N (2013) “Training changes processing of speech cues in older adults with hearing loss,“ *Front. Neurosci*, vol. 7, no. November, pp. 1–9, doi: 10.3389/fnsys.2013.00097 10.3389/fnsys.2013.00097 PMC3842592 24348347

[10] Connine CM, Mullennix J, Shernoff E, Yelen J (1990). Word Familiarity and frequency in visual and auditory Word Recognition. J Exp Psychol.

[11] Anderson S, Parbery-Clark A, Yi HG, Kraus N (2011). A neural basis of speech-in-noise perception in older adults. Ear Hear.

[12] Fleming D, Belleville S, Peretz I, West G, Zendel BR (2019). The effects of short-term musical training on the neural processing of speech-in-noise in older adults. Brain Cogn.

[13] Ciuffreda KJ (2002) “The scientific basis for and efficacy of optometric vision therapy in nonstrabismic accommodative and vergence disorders,“ *Optometry*, vol. 73, no. 12, pp. 735–762, 12498561

[14] Deekshita, Krishnan G (2019) “Lexical Database for High and Low Frequency Words,“

[15] Archana G, Krishna Y, Rajashekhar B (2017) “Efficacy of a novel structured aural rehabilitation module in kannada for adults with hearing impairment,“Manipal University,

[16] Yue WS, Azan N, Zin M (2013) “Voice Recognition and Visualization Mobile Apps Game for Training and Teaching Hearing Handicaps Children,“ *Procedia Technol*, vol. 11, no. Iceei, pp. 479–486, doi: 10.1016/j.protcy.2013.12.218

[17] Song JH, Skoe E, Banai K, Kraus N (2012) “Training to Improve Hearing Speech in Noise: Biological Mechanisms,” *Cereb. Cortex*, vol. 22, no. May, pp. 1180–1190, doi: 10.1093/cercor/bhr196 10.1093/cercor/bhr196 PMC3450924 21799207

[18] Stropahl M, Besser J, Launer S (2020). Auditory training supports Auditory Rehabilitation: a state-of-the-art review. Ear Hear.

[19] Nabelek AK, Letowski TR (1985) “Vowel Confusions of Hearing-imaired Listeners under Reverberant and nonreverberant Conditions,“ *J. Speech Hear. Disord*, vol. 50, no. May, pp. 126–131, 10.1044/jshd.5002.126 3990258

[20] Nabelek AK, Pickett JM (1974) “Monaural and Binaural Speech Perception through hearing aids under noise and reverberation with normal and hearing-impiared listeners,“Speech Percept., pp.724–739, 10.1044/jshr.1704.724 4444292

[21] Blamey P (2013). Factors affecting auditory performance of postlinguistically deaf adults using cochlear implants: an update with 2251 patients. Audiol Neurotol.

[22] Tye-Murray N “cLEAR Offers a Customized Approach to Aural Rehabilitation.“

[23] Burk MH, Humes LE (2008) “Effects of Long-Term Training on Aided Speech-Recognition Performance in Noise in Older Adults,“ *J. Speech, Lang. Hear. Res*, vol. 51, no. June pp. 759–771, 2008 10.1044/1092-4388(2008/054) PMC3179269 18506049

[24] Sweetow RW, Sabes JH (2010). Auditory training and challenges associated with participation and compliance. J Am Acad Audiol.

[25] Rubinstein A, Boothroyd A (1987) “Effect of Two Approaches to Auditory Training on Speech Recognition by Hearing Impaired Adults,“ *J. Speech Hear. Res*, vol. 30, no. June, pp. 153–160, 10.1044/jshr.3002.153 3599947

[26] TigerSpeech, Technology (2006) “Angel Sound.“

